# The effects of breathing exercises and inhaler training in patients with COPD on the severity of dyspnea and life quality: a randomized controlled trial

**DOI:** 10.1186/s13063-022-06603-3

**Published:** 2022-08-26

**Authors:** Yasemin Ceyhan, Pınar Tekinsoy Kartin

**Affiliations:** 1 Department of Internal Medicine, Kirsehir Ahi Evran University Faculty of Health Sciences Nursing Department, Bağbaşı Campus Faculty of Health Sciences 3rd Floor, Downtown, Kirşehir, Turkey; 2 Department of Internal Medicine, Erciyes University Faculty of Health Sciences Nursing Department, Kosk District, Kutadgu Bilig Street, Inside the Old Faculty, Melikgazi, Kayseri, Turkey

**Keywords:** COPD, Inhaler training, Breathing exercises, Dyspnea, Quality of life, Randomized controlled trial

## Abstract

**Background:**

Severe dyspnea and poor quality of life are common in chronic obstructive pulmonary disease (COPD). The most important reason for this is wrong applications in inhaler treatment. In addition, inhaler treatments that support non-pharmacological methods increase the effectiveness of the drug. The aim of this study was to determine the effects of breathing exercises and inhaler training for chronic obstructive pulmonary disease patients on the severity of dyspnea and life quality.

**Methods:**

The research was a randomized controlled trial. A total of 67 patients with COPD were included. The patients were randomized into two groups. Intervention group 1 were given pursed lip breathing exercise and inhaler training and Intervention group 2 were given only inhaler training. A follow-up after 4 weeks was carried out in both groups. Patient outcomes in both groups were assessed by a COPD assessment test (CAT), the Modified Medical Research Council (mMRC) scale, and the St. George’s Respiratory Questionnaire scale (SGRQ). This study followed the CONSORT checklist for randomized controlled trials. In the data analysis, independent *t*, Mann-Whitney *U*, ANOVA, Wilcoxon analysis, and Pearson chi-square tests were used.

**Results:**

The pursed lips exercise and inhaler drug use skills of patients in both groups increased (*p*<0.001). The median value of the CAT and mMRC scores were statistically significant for both groups (*p*<0.005). The mean of life quality scores of patients in both groups decreased, and this result was found to be statistically significant in all sub-dimensions and in the total scale score for both groups (*p*<0.001). Although the increase in the quality of life and the decrease in the severity of dyspnea of the patients in both groups were significant, neither group was superior to the other (*p*>0.05).

**Conclusions:**

As a result of the study, it was found that the skill of using the inhaler and the life quality of the patients increased, and the severity of dyspnea decreased. Supporting inhaler treatments with non-pharmacological methods can increase drug efficacy and quality of life.

**Trial registration:**

ClinicalTrials.gov NCT04739488. Registered on 21 Feb 2021.

## Background

Chronic obstructive pulmonary disease (COPD) is an important respiratory disease both in Turkey and in the rest of the world [1]. It is an important health problem that is not only a simple respiratory disease but also occurs with a combination of several underlying problems, in which airflow is restricted [2]. The fact that COPD affects more than one system over time causes difficulties in treatment and care, as well as an increase in deaths. COPD ranks third among the diseases that cause death both in Turkey and in the rest of the world [3, 4]. For this reason, it is extremely important to know the symptoms that occur in the patient and to be able to control them.

The most common symptom of COPD, which develops slowly and often occurs at greater ages, is dyspnea, which patients define as air hunger or shortness of breath [2, 3]. This condition is usually accompanied by cough, phlegm, wheezing, restriction of daily activities, fatigue, insomnia, and pain. Increased symptoms and restriction of daily activities also decrease the life quality of patients [3, 5–8].

The most important approach in relieving dyspnea and other symptoms is an accurate and regular pharmacological treatment [4]. The most effective pharmacological treatment is inhaler drug use, because the inhaler allows the drug to be delivered directly to the airways and causes fewer side effects compared to systemic treatment [7]. However, the only way to benefit from this effect of the inhaler is to use the drug correctly. In a systematic review in 2016, studies in the last 40 years were examined, and it was reported that inhaler drug misuse had increased greatly [9]. Similarly, it was found in many studies that patients used the inhaler with wrong techniques [9–11]. When the results of the studies were evaluated, it was seen that most of the errors on the use of the inhaler were related to breathing. Incomplete or incorrect steps such as failure of expiration before using the inhaler, not being able to inhale the drug at the appropriate flow rate and not holding the breath for the appropriate time after inhaling the drug suggested that the patients’ inhaler use should be supported by breathing exercises.

In addition to pharmacological treatment in dyspnea management, the use of non-pharmacological methods such as pursed lip breathing (PLB) leads to better airway patency and alveolar gas exchange for the patient and a decrease in dyspnea severity [12]. Particularly, PLB has been reported as B-level evidence in reducing the severity of dyspnea. The Canadian Thoracic Society Clinical Practice Guide emphasizes that the life quality of individuals should also be evaluated along with dyspnea in studies relating to COPD [7]. Considering all this, it is thought that patients need to use inhaler drugs in the correct steps and to support this use with PLB. However, there are no studies in the literature in which inhaler training supported by breathing exercise was applied. Therefore, this study was conducted to determine the effects of breathing exercises and inhaler training on the severity of dyspnea and life quality in COPD patients.

## Methods

### Study setting

The research was conducted at Ahi Evran University Training and Research Hospital in Kırşehir province in the Central Anatolia Region of Turkey.

### Type of study

The study was a randomized control trial and was conducted between September 2017 and December 2018.

### Ethical consideration

In order to carry out the study, approval was obtained from Kırşehir Ahi Evran University Training and Research Hospital (numbered 10670833/619 and dated 01 August 2017) and Kırşehir Ahi Evran University Faculty of Medicine Clinical Research Ethics Committee (numbered 2017/384 and dated 21 July 2017). In addition, verbal and written voluntary informed consent was obtained from the participants before starting the study.

### Randomization and participants

**Fig. 1:**
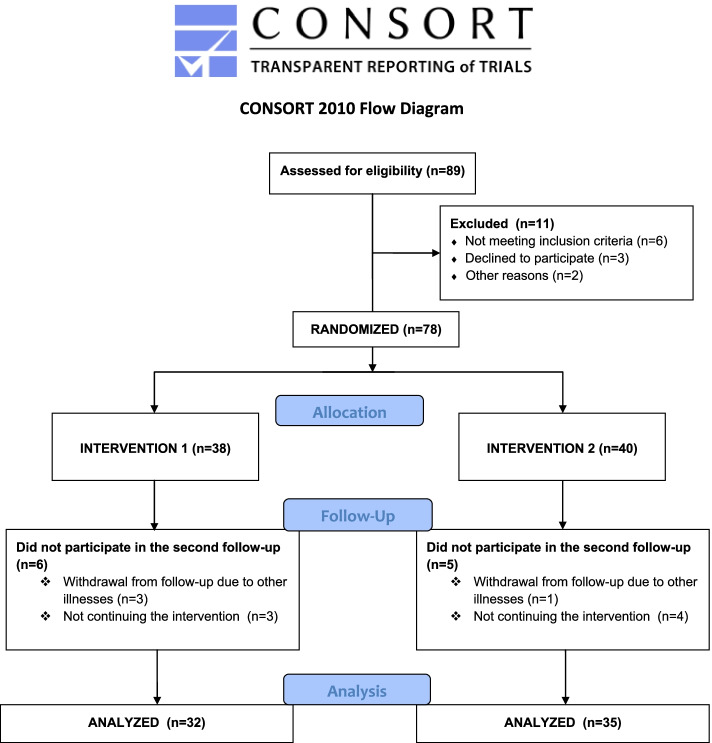
Sample diagram

All the COPD patients who applied to the chest diseases outpatient clinic of the hospital formed the population of the study. The patients were selected from among the volunteers who were monitored by the collaborating physician, who had applied to a single outpatient clinic and who met the inclusion criteria. The primary purpose of determining patient groups was to reduce bias and prevent patients from influencing each other. Therefore, the method of drawing lots was used. The draw was made by an independent observer other than the researchers. Firstly, Intervention 1 (I1) and Intervention 2 (I2) was written on two different papers, which were folded. The independent observer was asked to choose one of the papers. The paper he chose had I2 written on it. Therefore, all patients who came to the clinician examination that day were included in the I2 group. Those who came the next day were included in the I1 group. In this way, patients were recruited into groups on consecutive days. The patients included in the study are shown in the CONSORT flow diagram (Fig. 1).

Individuals were included in the study who were over the age of 18, who had been diagnosed with COPD for at least 3 months, who were using inhalers at least twice a day, who used their drug wrongly, who had not previously taken breathing exercise training, and who had not participated in a rehabilitation program [10, 11, 13, 14]. Individuals with mental disorders, communication disabilities, and heart disease that could lead to dyspnea and unstable angina were not included in the study.

### Sample size

Since there is no study similar to our study in the literature, the effect sizes suggested by Cohen were used to determine the effect size [15]. We utilized a within/between repeated measures analysis of variance. “Within” refers to expected differences between two time periods (first follow-up and second follow-up) and “between” refers to expected differences between the Intervention 1 and Intervention 2 groups.

The number of samples in the study was calculated as 27 in each group according to the values of estimated effect size *f*=0.25, type 1 error=0.05, power=0.95, number of groups=2, repetitions=2, correlation among repeated measures=0.5, and nonsphericity correction ɛ=1. The anticipated drop-out rate was 20%, and the study was completed with a total of 67 patients, 32 in Intervention I1 group and 35 in I2 group. The sample size was calculated using G*Power version 3.1.9.2.

The patients included in the study and their reasons for leaving are specified in the consort flow chart (Fig. 1). In the first step, 89 eligible COPD patients were identified. As a result of the interviews with the patients, it was understood that 11 patients did not meet the inclusion criteria and did not accept to participate in the study, and therefore were not included in the research. The first follow-up of the study was completed with 78 patients. Thirty-eight patients were in the I1 and 40 patients in the I2 group. However, some of these patients could not participate in the second interviews due to the reasons stated in Fig. 1, and these patients were also excluded from the study.

### Intervention protocol

Firstly, the questionnaire forms were given on the first day when the patients were included in the study. After completing the form, the patient was instructed in accordance with the intervention group. In accordance with the training content, patients were asked to continue the procedures they had learned twice a day for four weeks. In addition, the researchers provided consultancy by calling the patients twice a week. After the intervention was continued for 4 weeks, the patients were invited for re-evaluation to the hospital, where the second follow-up of patients who completed the 4-week procedure was performed. In this follow-up, the questionnaires were given again and the patients’ inhaler use skills were evaluated.

#### Intervention 1 group

In the first interview, sociodemographic information was obtained from the patients, and their COPD, levels of dyspnea and life quality, and their skill of using the inhaler were evaluated. Patients who used their drug wrongly were given training, and PLB exercise was taught. In the interview room of the outpatient clinic, the researcher first applied the PLB exercise herself and then repeated the application steps for the patient by both explaining and showing them until they had learned. Then, the application was made together with the patient and the points that the patient was unable to do were corrected. After the application, the patient was rested and the training of inhaler drug use was started.

The researchers used a placebo drug specifically for each type of inhaler used by the patient to provide training by the demonstration method. The patient was also allowed to repeat the application with a different placebo drug, and the training was continued until the incorrect steps were corrected. At the end of the training, the patients were given leaflets enriched with color pictures, which they could read at home to remember the steps they had forgotten.

#### Intervention 2 group

In the first interview, the patients’ sociodemographic information was obtained and their COPD, levels of dyspnea and life quality and their skill of using the inhaler were evaluated. Then, each patient was taught the correct application steps according to the type of inhaler he or she used as described above. The difference of this group from group I1 was that the patients were given only training on inhaler drug use, and PLB exercise was not given. At the end of the training, inhaler drug use brochures were given.

### Data collection

#### Forms prepared by the researchers

These were a Patient Information Form, an Inhaler Use Skill Chart, and a Breathing Exercise and Inhaler Use Skill Chart [10, 11, 14, 16]. The *Patient Information Form* consisted of 19 questions including the sociodemographic and disease features of the patients. The *Breathing Exercise and Inhaler Application Skill Chart* consisted of 18 items including the steps of PLB and inhaler use to be applied to group I1. The *Inhaler Application Skill Chart* consisted of 10 items including only the skill of using inhaler, to be applied to group I2. Correct steps were evaluated as 1 point and wrong steps as 0 points in both skill charts.

#### COPD Assessment Test (CAT)

This scale, developed by Jones et al. [16], is used to measure the health status of individuals with COPD. There are 8 questions in total in the scale, which is scored as an increasing Likert scale between 0 and 5. The Turkish validity and reliability study of the scale was conducted by Yorgancıoglu et al. [17] and reported to be appropriate. The Cronbach’s *α* coefficient of the scale in this study was calculated as 0.95 in the first follow-up and the last follow-up, and it was found to be highly reliable.

#### St. George’s Respiratory Questionnaire (SGRQ)

This is a quality of life questionnaire specific to patients with COPD, developed by Jones and Forde [18]. There are 50 questions in total in the scale, which is scored as an increasing Likert scale between 0 and 5. The Turkish validity and reliability study of the scale was conducted by Polatlı et al. [19] and reported to be appropriate. Cronbach’s *α* coefficient in this study was calculated as 0.84 in the first follow-up and 0.88 in the second follow-up, and the scale was found to be highly reliable.

#### Modified Medical Research Council (mMRC) Scale

This was developed by the British Medical Research Council in order to provide information about the degree of dyspnea experienced by the patients with COPD based on their and their perception of the disease [20]. There are 5 questions in total in the scale, which is point as between 0 and 4. It has been stated that the scale can be used safely in the evaluation of dyspnea in studies conducted in Turkey [21]. As it is a one-dimensional scale, the Cronbach's *α* coefficient could not be calculated.

### Data analysis

Data were evaluated with IBM SPSS Statistics 25.0 (Statistical Package for the Social Sciences; IBM Corp., Armonk, New York, ABD) and MINITAB statistical program package. After evaluations were made with the Shapiro Wilk normality test, the two-sample independent *t*-test was used for normal distribution, and the Mann-Whitney *U* test was used for non-normal variables. Comparisons of groups over time were made with two-way analysis of variance in repeated measurements for variables with normal distribution. Categorical variables were included in the model with dummy coding. Bonferroni correction was applied, and comparisons of the main effects and intragroup comparisons for variables that did not show normal distribution were made with Wilcoxon analysis. The relationship between categorical variables was examined with the exact method of the Pearson chi-square test. A value of *p*<0.05 was considered statistically significant in the study. Reliability measurements of the scales were made according to Cronbach's α coefficient.

## Results

It was found that 56.3% of group I1 were between the ages of 60 and 69, 56.3% had been diagnosed with COPD for 1-4 years, and 90.6% of these patients described their most common problem as shortness of breath. In group I2, 48.6% of the patients were between the ages of 60 and 69, 48.6% had been diagnosed with COPD for 1–4 years, and 82.9% of the patients reported that the problem they complained about most was shortness of breath. No statistical difference was found between groups I1 and I2 in terms of descriptive features (*p*>0.05) (Table 1).

**Table 1:** Distribution of introductory features of Intervention 1 and Intervention 2 groups

	Group						
Introductory Features	Group I1 (*n*=32)		Group I2 (*n*=35)		Total (*n*=67)		Test *p*
	*n*	%	*n*	%	*n*	%	
**Age group**							
40–49 years	1	18.8	2	5.7	3	4.5	0.937
50–59 years	6	3.1	8	22.9	14	20.9	
60–69 years	18	56.3	17	48.6	35	52.2	
70 years and older	7	21.8	8	22.8	15	22.4	
**Gender**							
Female	3	9.4	4	11.4	7	10.4	1.000
Male	29	90.6	31	88.6	60	89.6	
**Educational status**							
Illiterate/primary school	21	65.6	28	80.0	49	73.0	0.107
Secondary school/high school	8	25.0	4	11.4	12	18.0	
Associate degree/bachelor’s degree	3	9.4	3	8.6	6	9.0	
**Marital status**							
Married	31	96.9	33	94.3	64	95.5	1.000
Single	1	3.1	2	5.7	3	4.5	
**Time since diagnosis**							
1–4 years	18	56.3	17	48.6	35	52.2	0.763
5–9 years	5	15.6	8	22.9	13	19.4	
10 years +	9	28.1	10	28.6	19	28.4	
**Smoking status**							
Smoker	5	15.6	9	25.7	14	20.9	0.598
Ex-smoker	24	75.0	24	68.6	48	71.6	
Non-smoker	3	9.4	2	5.7	5	7.5	
**Alcohol drinking status**							
Ex-drinker	9	28.1	9	25.7	18	26.9	0.885
Non-drinker	23	71.9	26	74.3	49	73.1	
The most common problems*							
Shortness of breath	29	90.6	29	82.9	58	86.6	0.480
Cough	19	59.4	24	68.6	43	64.2	0.433
Phlegm	16	50.0	23	65.7	39	58.2	0.223
Fatigue	10	31.3	7	20.0	17	25.4	0.401
Insomnia	8	25.0	6	17.1	14	20.9	0.551
Wheezing	8	25.0	12	34.3	20	29.9	0.437
Sweating	4	12.5	10	28.6	14	20.9	0.138
**Hospitalization status (last 1 year)**							
Yes	13	40.7	10	28.6	24	35.8	0.763
No	19	59.3	25	71.4	43	64.2	
**Duration of hospital stay**							
1-4 days	6	46.2	4	40.0	10	43.5	1.000
5 days +	7	53.8	6	60.0	13	56.5	
Type of inhaler**							
MDI	24	75.0	26	74.2	50	37.3	**
Diskus/Discair/Sanohaler	14	43.7	19	25.7	33	24.6	
Neohaler/Aerolizer	14	43.7	12	34.2	26	19.4	
Handihaler	12	37.5	13	37.1	25	18.6	
**Need to use an extra inhaler during the day**							
Yes	12	37.5	16	45.7	28	41.8	0.806
No	20	62.5	19	54.3	39	58.2	
**Training status for inhaler utilization**							
Trained	18	58.1	26	74.3	44	66.7	0.197
Not trained	13	41.9	9	25.7	22	33.3	

*Patients gave more than one answer

**Some patients use more than one inhaler so no comparison could be made

It was observed that most of the patients made errors in all steps of the breathing exercise in the first follow-up. These errors were observed as inability to breathe through the nose at the appropriate time, inability to perform expiration in twice the time of inspiration and with appropriate force, inability to purse the lips as if whistling while exhaling, and inability to continue the application for 10 min. After the training, it was found that most of the steps had been learned, and the difference between the two follow-ups was significant (*p*<0.001) (Table 2).

**Table 2:** Breathing exercise skills of patients in the intervention 1 group at the first and last follow-up

Steps of breathing exercise	I1 Group (*n*=32)				p*
	Yes		No		
	First follow-up	Last follow-up	First follow-up	Last follow-up	
Sit comfortably and breathe through your nose for 2–3 s like smelling flowers	3	32	29	0	**<0.001**
Purse your lips like whistling and exhale slowly.	4	32	28	0	**<0.001**
Try to exhale from just your lips in 4–6 s	0	31	32	1	**<0.001**
Exhale like blowing the flame of a candle but not extinguishing it.	1	31	31	1	**<0.001**
Do not inflate your cheeks and do not tighten your abdominal muscles while exhaling	2	31	30	1	**<0.001**
Take a normal comfortable breath after 2 or 3 applications in a row	0	32	32	0	**<0.001**
Continue this exercise for about 10 min, but rest when you have difficulty	0	32	32	0	**<0.001**
Rest for 10 min after the exercise and move on to the drug application steps	0	31	32	1	**<0.001**

*McNemar and two-proportions *z* test

When the inhaler drug use steps of the patients in groups I1 and I2 in the study were examined, it was seen that the errors made before the training were quite high in both groups. It was observed that most of the errors were in steps such as not exhaling before applying the drug, not being able to apply hand-breath coordination, not being able to inhale at the appropriate speed, not being able to hold the inhaled drug for 10 s, and not performing a mouthwash after using the inhaler. It was found that these steps were learned after the training and the difference between the two follow-ups was significant (*p*<0.001).

The mean scores of the patients received from the inhaler types increased in the last follow-up compared to the first follow-up, and this increase was found to be statistically significant in I1 (*p*<0.001, *p*<0.001, *p*<0.001, *p*=0.002) and in I2 (*p*<0.001, *p*<0.001, *p*=0.003, *p*=0.002). The differences between the inhaler scores of the patients in groups I1 and I2 at the first and last follow-up were similar in all types of inhaler and no statistical significance was found between the differences (*p*>0.05). This shows that the training given to the groups was the same. The distribution of inhaler types was examined according to the first follow-up of the individuals in groups I1 and I2, and the two groups were found to be similar in this respect (*p*>0.05) (Table 3).

**Table 3:** Distribution of inhaler score differences of patients in intervention 1 and intervention 2 groups

Intervention Groups and Tests		Inhaler drug types			
		MDI	Diskus/Discair/Sanohaler	Neohaler/Aerolizer	Handihaler
**Intervention 1 group** (*n*=32)	**First follow-up** *M* (*Q*_1_–*Q*_3_)	6.0 (5.0–7.0)	6.0 (5.5–8.0)	6.0 (5.5–7.5)	6.5 (5.25–7.0)
	**Last follow-up** *M* (*Q*_1_–*Q*_3_)	10.0 (10.0–10.0)	10.0 (10.0–10.0)	10.0 (10.0–10.0)	10.0 (10.0–10.0)
	Difference* *M* (*Q*_1_–*Q*_3_)	4.0 (5.0–3.0)	4.0 (4.5–1.5)	4.0 (4.5–2.5)	3.5 (4.75–3.0)
	p**	**<0.001**	**<0.001**	**<0.001**	**0.002**
**Intervention 2 group** (*n*=35)	**First follow-up** *M* (*Q*_1_–*Q*_3_)	5.0 (4.0–7.0)	6.0 (5.0–7.0)	6.5 (6.0–8.0)	6.0 (5.5–7.5)
	**Last follow-up** *M* (*Q*_1_–*Q*_3_)	10.0 (9.0–10.0)	10.0 (9.0–10.0)	10.0 (9.0–10.0)	10.0 (9.0–10.0)
	Difference* *M* (*Q*_1_–*Q*_3_)	5.0 (5.0–3.0)	3.0 (4.0–3.0)	3.0 (3.75–2.0)	3.0 (4.5–2.5)
	p**	**<0.001**	**<0.001**	**0.003**	**0.002**
**I1 and I2 group difference comparison** p***		0.464	0.953	0.173	0.390
**I1 and I2 group first follow-up comparison** p***		0.306	0.632	0.460	0.932

*M* median, *Q*_*1*_ 25th percentile, *Q*_*3*_ 75th percentile

*The difference was obtained by subtracting the first follow-up score from the last follow-up score

**Since the data are not parametrically distributed, z: Wilcoxon analysis was used

***Since the data are not parametrically distributed, z: Mann-Whitney *U* test was used

The median value of the CAT score difference of the patients in group I1 decreased from 35.5 in the first follow-up to 27.0 in the last follow-up and from 34.0 to 29.0 in group I2. This decrease indicated that the patients’ COPD assessment status was improving, and the result was statistically significant for both groups (*p*<0.001). The difference between the total CAT score in the patients’ first and last follow-up in I1 and I2 was found to be 5.5 in I1 and 3.0 in I2, and the result was statistically significant (*p*<0.05). The median value of the mMRC scale score difference of the patients in groups I1 and I2 decreased from 4.0 at the first follow-up to 3.0 at the last follow-up. This situation revealed that the severity of dyspnea in patients decreased and the result was statistically significant for both groups (I1=*p*<0.001, I2=*p*<0.05). The differences between the mMRC scores of the patients in groups I1 and I2 at the first and last follow-up was found to be 1.0 in I1 and 0.0 in I2, and the result is statistically significant (*p*<0.05) (Table 4). Thus, it was concluded that breathing exercises and inhaler training applied twice a day for 4 weeks to patients with COPD reduces the severity of dyspnea.

**Table 4:** Distribution of score differences with COPD assessment and mMRC scores of the patients in the Intervention 1 and Intervention 2 groups at the first and last follow-up

Intervention groups and tests		CAT	mMRC
**I1 group** (*n*=32)	**First follow-up** *M* (*Q*_1_–*Q*_3_)	35.5 (30.25–38.75)	4.0 (3.0–4.0)
	**Last follow-up** *M* (*Q*_1_–*Q*_3_)	27.0 (21.0–32.5)	3.0 (3.0–3.0)
	Difference* *M* (*Q*_1_–*Q*_3_)	5.5 (4.0–9.0)	1.0 (0.0–1.0)
	p**	**<0.001**	**<0.001**
**I2 group** (*n*=35)	**First follow-up** *M* (*Q*_1_–*Q*_3_)	34.0 (28.0–36.0)	4.0 (3.0–4.0)
	**Last follow-up** *M* (*Q*_1_–*Q*_3_)	29.0 (20.0–34.0)	3.0 (3.0–4.0)
	Difference* *M* (*Q*_1_–*Q*_3_)	3.0 (2.0–5.0)	0.0 (0.0–1.0)
	p**	**<0.001**	**0.001**
**Difference comparison of groups I1 and I2** p***		**0.002**	**0.040**

*M* median, *Q*_*1*_ 25th percentile, *Q*_*3*_ 75th percentile

*The difference was obtained by subtracting the first follow-up score from the last follow-up score

**Since the data are not parametrically distributed, z: Wilcoxon test was used

***Since the data are not parametrically distributed, z: Mann-Whitney *U* test was used

The distribution of life quality scores of the patients in groups I1 and I2 at the first and last follow-up is given in Table 5 by correcting for all covariates. The mean of life quality scores of patients in both groups decreased in effect, symptom, activity, and overall score of the scale from first follow-up to last follow-up. As the score obtained from the quality of life scale decreases, the life quality of the patients increases. Accordingly, in sub-dimensions and in the total, life quality level of the patients in groups I1 and I2 increased compared to their first follow-up. This result was found to be statistically significant in all sub-dimensions and in the total scale score for the two groups (time effect *p*<0.001) (Table 5).

**Table 5:** Distribution of life quality scores of the patients in Intervention 1 and Intervention 2 groups at the first and last follow-up

SGRQ sub-dimensions and total score		Groups		*Test statistic* p**
		Intervention 1 (*n=*32) $\overline{x}\pm sem$	Intervention 2 (*n=*35) $\overline{x}\pm sem$	
**Effect**	**First follow-up**	51.89±3.27	50.04±3.1	*F=*0.158 *p=*0.693
	**Second follow-up**	29.47±3.67	33.5±3.5	*F=*0.606 *p=*0.440
	*Test statistic* p**	*F=*87.035 *p<***0.001**	*F=*51.889 *p<***0.001**	
	*Group × time effect: *F=*2.989; p*=0.090; **group effect:** *F*=0.057; *p=*0.812; **time effect:** *F*=8.876; *p=***0.004**			
**Symptom**	**First follow-up**	65.23±2.87	63.25±2.74	*F=*0.232 *p=*0.632
	**Second follow-up**	60.45±2.91	60.14±2.77	*F=*0.006 *p=*0.937
	*Test statistic* p**	*F=*38.552 *p<***0.001**	*F=*18.209 *p<***0.001**	
	*Group × time effect: *F=*2.261; *p=*0.139; **group effect:** *F*=0.079; *p=*0.779; **time effect:** *F*=6.114; *p=***0.017**			
**Activity**	**First follow-up**	72.57±2.97	70.53±2.84	*F=*0.233 *p=*0.631
	**Second follow-up**	56.69±3.32	56.39±3.17	*F=*0.004 *p=*0.949
	*Test statistic* p**	*F=*40.701 *p<***0.001**	*F=*35.496 *p<***0.001**	
	*Group × time effect: *F=*0.241; *p=*0.626; **group effect:** *F*=0.081; *p=*0.777; **time effect:** *F*=17.919; *p* ***<*0.001**			
**Total**	**First follow-up**	60.37±2.71	58.44±2.59	*F=*0.249 *p=*0.620
	**Second follow-up**	42.87±3.01	44.88±2.87	*F=*0.220 *p=*0.641
	*Test statistic* p**	*F=*83.798 *p<***0.001**	*F=*55.362 *p<***0.001**	
	*Group × time effect: *F=*2.093; *p=*0.154; **group effect:** ***F*=0.001;** ***p=*0.991; time effect:** *F*=14.257; *p <***0.001**			

***F***, two-way repeated measures Anova

*Group **×** time effect, the comparison value between groups of the first and last follow-up differences of each group

**Adjusted for age, gender, educational status, marital status, time of diagnosis, smoking status, alcohol drinking status, hospitalization status (last 1 year), duration of hospital stay, and training status for inhaler utilization

When the effect of training on the groups was examined, it was found that the groups did not have superiority over each other in sub-dimensions or total scale score, and this did not create a statistically significant difference (group effect *p*>0.05). When the effectiveness of training on time and among the groups of the patients in groups I1 and I2 was analyzed, it was seen that there was no statistically significant difference in sub-dimensions or total scale score (group × time effect *p*>0.05). Thus, considering the time effect of group I1, it was found that it did not have any superiority over group I2.

## Discussion

Although correct inhaler utilization is extremely important in reducing complaints that can be experienced by COPD patients, many studies have revealed that a lot of patients use inhalers wrongly [9–11, 22]. In this study, similar to the literature, it was found that patients made a lot of errors in all types of inhaler. As a result of the failure of effective expiration before using the drug, which is the leading cause of the errors, drug particles do not remain in the airways [23]. Göriş et al. [10] and Özel et al. [22] reported that expiration was not performed before using the drug. In addition, the drug must be inhaled at an appropriate flow rate in order for the drug particles to reach the airways in the periphery [23]. Takaku et al. [11] showed in a study in 2017 that the flow rate of the drugs was not appropriate. In this study, similar to the literature, it was found that patients did not expire before using the inhaler and were not able to inhale at the appropriate flow rate.

Retaining the drug by holding the breath for a certain period of time after the application of the drug is an important step which is necessary for of the drug particles to settle in the airways. A time of approximately 10 s is suggested [24]. In this study, it was observed that the patients did not pay attention to this step at the first follow-up, but they held their breath for a suitable period after training.

When the differences in scores were examined according to the inhaler types used by the patients, it was observed that the lowest score difference in group I1 was in the handihaler group (Table 3). It is thought that this is due to the fact that the patients made the most errors in the steps related to breathing during the first follow-up, and the breathing exercises given to group I1 were effective. In addition, it has been supported by many studies that the handihaler, which is dry powder inhaler type, is easier to use than the other inhaler types [10, 11, 22]. According to the first follow-up comparison test in Table 3, the groups were similar in terms of the inhaler types used (*p*>0.05). In the same way, the fact that the I1 and I2 group difference comparison test was not significant in all inhaler types means that the training given was similar (*p*>0.05).

It has been reported in the literature that breathing exercises can increase the volume that patients will inhale before inhaler drug use [23]. In particular, it has been reported that PLB will reduce dyspnea and the number of breaths per minute, providing ventilation efficiency so that inhaler drugs will perform better [12]. In this study, the patients in group I1 were asked to perform PLB at least twice a day for 10 min. Initially, only a few of the patients who did not have any knowledge of breathing exercises were seen to try with their own effort to breathe out as if they were whistling when their symptoms increased, but it was understood that they could not perform the procedure correctly. It was determined at the end of the training that PLB had been learned. It has been reported in the literature that PLB exercise provides various benefits for patients with COPD. Decrease in severity of dyspnea and the number of ventilations per minute and an increase in oxygen saturation and exercise capacity are among these benefits [14, 25]. In this study, it was observed that patients in group I1 who were taught PLB scored better in CAT (*p*=0.002) and mMRC dyspnea severity (*p*=0.040) than group I2 (Table 4).

While the symptoms caused by COPD affect the daily work of an individual, they also determine the perception of the disease. Problems created by COPD in patients were evaluated with CAT. It was found that the CAT score of both groups decreased and the results were significant (p<0.001). When the score differences between the groups were examined, the greater effect in group I1 revealed that breathing exercise performed in addition to inhaler training contributed positively to COPD assessment (Table 4). Similarly, in the literature [26, 27], it is reported in studies involving inhaler training and breathing exercises given to patients with COPD that the total CAT score of the intervention group patients decreased and the result was significant compared to the control group (*p*<0.05).

The effect of trainings on the dyspnea severity of patients was evaluated by the mMRC scale, and it was revealed that the training given was effective in group I1 (*p*<0.001) and group I2 (*p*=0.001). According to the score differences between the groups, the effect in group I1 was higher, and it was found that breathing exercise applied in addition to inhaler training contributed positively to the perception of dyspnea (Table 4). In the literature [7, 10, 12, 26, 28, 29], many studies reporting that the training given to patients with COPD contributed positively to the severity of dyspnea support the results of this study.

Airway obstruction and accompanying symptoms in COPD have a negative effect on the quality of life [7, 8]. The effect of the training given in the study to the patients on the quality of life was examined, and it was seen that there were improvements in both groups in the sub-dimensions and overall score of the quality of life scale at the last follow-up compared to the first follow-up. Reports in other studies conducted with COPD patients that the training given improves the quality of life [7, 10, 29–31] are similar to the quality of life findings of this study.

In the present study, quality of life did not make any difference in terms of training given to groups I1 and I2. It was expected according to the literature that PLB exercise given to group I1, unlike group I2, would be significant in terms of quality of life [5, 7, 10]. In a study conducted by Dogan [27] with planned training given to patients with COPD, it was reported that after PLB training, the scores on the sub-dimensions of the quality of life scale and the total scale score of the intervention group decreased, and quality of life increased. Similarly, in this study, it was seen that the total quality of life score of group I1, which included patients given PLB training, was better than that of group I2. However, there was no statistical significance between the two groups in terms of the quality of life scale (Table 5). Some features of group I2 in the study were thought to affect this significance. Especially, the longer time since diagnosis of group I2 shows that the patients adapt better to COPD. Studies have reported that as the time since diagnosis increases, learning to live with the disease can be positively affected [10, 32]. In addition, the statements of the patients in group I2 that they received more inhaler training when they were first diagnosed, that they stayed less in the hospital in the last year and that their stay was shorter, and that they experienced fewer symptoms such as shortness of breath, fatigue, and insomnia are factors that may cause a difference between the two groups. The increase in shortness of breath, which is one of the important symptoms of COPD, brings with it the symptoms of insomnia and fatigue. These symptoms affect the patient significantly and decrease the quality of life [3, 8, 33].

The feeling of shortness of breath in particular is an important factor that affects the daily routine and care actions of an individual, ultimately decreasing their quality of life. Demir et al. [5] examined the relationship between dyspnea and quality of life and reported that the quality of life decreased as the severity of dyspnea increased. In a systematic review, Geddes et al. [34] reported that the quality of life increased with a decrease in the severity of dyspnea in COPD patients, but that more detailed studies were needed to provide an evidence-level thesis. In this study, the fact that the dyspnea level of the patients was found to be high and quality of life was low showed similarity with the literature.

## Conclusions

According to the results of the study, it was found that PLB exercise and inhaler training applied to patients with COPD improves breathing exercise and inhaler using skills, reduces the negative effects of COPD on the individual, relieves the severity of dyspnea, and improves the quality of life. In line with these results, the following measures can be suggested: training for inhaler drug use by nurses, teaching non-pharmacological methods such as PLB exercise, the support of nurses for patients with evidence-based practices in COPD by following the current literature, establishing special units under the leadership of COPD nurses in hospitals, conducting regular patient interviews in hospitals, and supporting the research results by planning a larger sample and with a longer time interval.

### Limitations

The limitations of the study are that patients with COPD living outside the city center were not included in the study due to low possibility for follow-up, that verbal statements that the patients applied the training they received correctly were accepted, and that the patients could not be evaluated in the long term as the study lasted only 4 weeks. In addition, the research is limited only to the group in which it is conducted and cannot be generalized.

## Data Availability

Applicable.

## Abbreviations

COPD: Chronic obstructive pulmonary disease
CAT: COPD Assessment Test
mMRC: Modified Medical Research Council
SGRQ: St. George’s Respiratory Questionnaire
CONSORT: Consolidated Standards of Reporting Trials
PLB: Pursed lip breathing
